# Promoting the recruitment of historically underrepresented children and families in clinical trials: Perspectives of pediatric clinic staff

**DOI:** 10.1016/j.conctc.2026.101607

**Published:** 2026-01-27

**Authors:** Janvi D. Nanavati, Daniel Mendoza Martinez, Grace W. Ryan, Melissa Goulding, Sonia Radu, Michelle Spano, Ted Kremer, Kali Pereira, John Almeida, Christine Frisard, Sybil Crawford, Milagros C. Rosal, Nancy Byatt, Stephenie C. Lemon, Lori Pbert, Michelle Trivedi

**Affiliations:** a Division of Pulmonary Medicine, Department of Pediatrics, University of Massachusetts Chan Medical School, 55 N Lake Ave, Worcester, MA, 01605, USA; b Department of Population and Quantitative Health Sciences, University of Massachusetts Chan Medical School, 55 N Lake Ave, Worcester, MA, 01605, USA; c Tan Chingfen College of Nursing, University of Massachusetts Chan Medical School, 55 Lake Ave N, Worcester, MA, 01605, USA; d Lifeline for families Center, Department of Psychiatry, University of Massachusetts Chan School of Medicine, 55 N Lake Ave, Worcester, MA, 01605, USA; e Dept of Obstetrics and Gynecology, University of Massachusetts Medical School, 55 N Lake Ave, Worcester, MA, 01605, USA; f Child Health Equity Center, Department of Pediatrics, UMass Chan Medical School, UMass Memorial Children's Medical Center, 55 N Lake Ave, Worcester, MA, 01605, USA

**Keywords:** Clinical trial recruitment, Pediatrics, Health equity, Qualitative research

## Abstract

**Introduction:**

Children and families from historically marginalized ethnic/racial backgrounds have low participation in clinical trials and pediatric practice staff perspectives on this topic are underexplored.

**Methods:**

We conducted interviews (n = 20) with pediatric practice staff and used rapid template analysis to identify themes and sub-themes.

**Results:**

We identified several primary themes related to strategies that both research staff and pediatric practice staff can use in order to support the recruitment of historically marginalized populations into research. For example, researchers can facilitate running reports within practices to identify potential trial participants with limited access to care and pediatric providers can offer research opportunities at well visits noting potential benefits of research and directly discuss mistrust in research.

**Discussion:**

While the dynamics involved in the recruitment of historically marginalized children and families into trials are inherently complex, we identified several concrete strategies to support this work and increase diversity in pediatric clinical trials.

## 1 Introduction

Clinical trials are the gold standard for generating safe and effective health interventions for children and families [1,2]. However, low participation rates among underrepresented and marginalized populations in clinical trials is a persistent problem; for example the literature shows that over 66 % of participations in pediatric clinical trials are white, with very low participation from historically marginalized populations (12 % Black, 7 % Hispanic) [3] and reports describe that a mere 25 % of trial participants identified as non-white [4]. This contributes to persistent health inequities in minoritized populations that experience higher levels of morbidity from chronic diseases [1,3,[5], [6], [7]]. Pediatric practices are a logical place to promote the recruitment of diverse participants, and pediatric providers are well-positioned to support the recruitment of historically marginalized children and families [[8], [9], [10], [11]]. Pediatricians are trusted healthcare providers that have long-term relationships with children and families [12,13]; thus they are potentially trusted messengers to present clinical trial opportunities [14]. There is strong evidence that the inclusion of provider and practice is critical to addressing complex real-world problems related to research [15], and diverse perspectives can help us understand how to improve representation in pediatric trials. However, we know little about perspectives of pediatric providers and clinic staff who participate in clinical trial recruitment. Barriers to recruiting historically marginalized adult populations are well-described (e.g., time, language discrepancies, and lack of post-study continuity of care) [16], yet barriers to recruiting historically marginalized children and families have been less explored [17].

We were able to examine these issues by interviewing pediatric clinic staff involved in our pilot cluster randomized control trial of Asthma Link, a school supervised therapy intervention. In the Asthma Link pilot trial, pediatric providers helped to recruit a high percentage of historically marginalized children and families from the pediatric practice setting. Seventy-five percent of the parent-child dyads participating in the Asthma Link pilot identified as low-income with substantial representation from historically minoritized racial/ethnic backgrounds (65 % Hispanic; 30 % Black) [18]. The objective of our study was to elicit the perspectives of pediatric practice staff who supported recruitment for a community-based pediatric clinical trial wherein many recruited participants were parent-child dyads from low-income and historically marginalized racial/ethnic backgrounds.

## 2 Methods

We conducted semi-structured interviews with pediatric practice staff who had previously supported recruitment for the pilot trial of Asthma Link (CT.gov number: [Blinded for review]), which is a school-supervised asthma therapy intervention wherein children with poor medication adherence receive their daily asthma medication under school nurse supervision. The Asthma Link protocol has been previously described [19,20]. All five pediatric practices that participated in pilot testing of Asthma Link, serve historically marginalized and low-income populations. All staff members from these five clinics who had previously participated in Asthma Link recruitment were eligible to participate (n = 29). We invited potential participants via individual emails and followed up a maximum of five times.

With the goal of improving engagement and recruitment of children and families from low-income and historically marginalized backgrounds into research, we designed a flexible semi-structured interview guide to elicit pediatric practice staff perspectives 1) communication with families about clinical trial recruitment, 2) perceived motivators, barriers, and facilitators for families to participate in research, and 3) motivators, barriers, and facilitators for pediatric practice staff to participate in research. (See supplemental materials). The guide contained 8 questions, many of which had structured probes or follow-up questions and the interviewers were trained to ask probing questions to elicit additional details on participant responses.

The web-based interviews were conducted by 3 of the authors (GWR, DMM, JDN). Two interviewers were medical students (DMM, JDN) trained by a qualitative research expert (GWR). The students were trained comprehensively in qualitative interview conduct and analysis, including considerations of reflexivity [21]. The interviews were recorded and transcribed using an automated transcription feature, thus field notes were not taken. All transcripts were reviewed and edited for accuracy by a member of the research team. The interviewers did not have any prior relationships with the participants. The University of University of Massachusetts Chan Medical School Institutional Review Board reviewed and approved all study procedures described.

We used rapid qualitative template analysis to analyze interview data [22]. The analysis team used a deductive approach, building from the interview guide, to create a template which was used to summarize interviews. Briefly, for each interview guide question, we created a representative domain name. For example, for the question “Can you tell me a little bit about how you had the conversation with families about the study?”, the domain was named “recruitment communication.” These domains helped us to organize summaries within the templates. Once the template was created, the analysis team used it to summarize two interviews independently and then met to compare those summaries to ensure consistency. After one round of this group summarizing, all discrepancies in applying the template were resolved through discussion among the three analysts, led by the senior analysts. We primarily identified discrepancies in the breadth and depth of summaries and through group discussion determined the level of detail needed to include in remaining summaries. After this consensus discussion, the remaining interviews were summarized by one interviewer. The analysis team met biweekly to review summaries and discuss progress towards thematic saturation, which was defined as the point at which no new themes emerged [23]. The resulting summaries were combined to create a matrix with all domains included, identifying themes and subthemes. These were defined as recurrent topics that practice staff felt necessary to address, with larger themes coming up in almost every interview and some subthemes more commonly encountered than others. We concluded interviews when thematic saturation was achieved [24].

## 3 Results

Of the 29 potential participants invited, we conducted interviews with 20 pediatric practice staff. The nine individuals that did not participate were unable to be reached due to scheduling challenges or non-response by email. The interviews averaged 15 min (ranged 8–28 min) and participant demographics are presented in Table 1. We identified two primary themes described below and Table 2 presents these themes along with sub-themes, and representative quotations.

**Table 1:** Participant characteristics: Pediatric providers and practice staff (n = 20).

Demographics	Categories	N(%)/Median (Interquartile range)
Professional Title	Pediatricians, Attendings	12 (60.0)
	Pediatric Residents	2 (10.0)
	Nurse Practitioners/Physicians Assistants	3 (15.0)
	Practice Managers	3 (15.0)
Type of Medical practice	Community Practice	12 (60.0)
	Academic Practice	8 (40.0)
Race/ethnicity	White	13 (65.0)
	Indian/South Asian	4 (20.0)
	African American	1 (5.0)
	Asian	1 (5.0)
	Middle Eastern	1 (5.0)
Gender	Male	3 (15.0)
	Female	17 (85.0)
Years of experience	Pediatricians, Attendings	16.4 (2.5–45)
	Pediatric Residents	1 (1,1)
	Nurse Practitioners/Physicians Assistants	12.3 (2–20)
	Practice Managers	23 (21–25)

**Table 2:** Pediatric clinic staff perspectives on clinical trial recruitment of diverse pediatric populations.

Themes	Subthemes	Representative Quotations
*Approaches that pediatric practice staff can use to support recruitment of historically marginalized children and families into clinical trials*	Openly discussing with families the potential mistrust in research to help build trust in research processes	“You know there's oftentimes a mistrust that's there and you know inherently and so in science and particularly with studies that have taken advantage of racialized populations in our country. So I think there's probably a hurdle, like conscious or subconscious for many minorities families engage in.” (Provider)
		“Again, we deal with, you know, a very large Medicaid population. And so they're always concerned is DCF [going to] get involved? If I answer one of these questions wrong, are you [going to] report it to DCF? Does it make me a bad parent?” (Practice Manager)
	Discussing potential clinical benefits and financial compensation	“It's keeping kids healthy, but they're also getting something [financial incentive] out of it. I mean, as much as I hate to say that, but I think the family is getting something out of it, It pushes them more to want to do it” (Provider) “I mean, we explained it to them, what the asthma study was and how that is going to help the child to manage their treatment and the education piece of it which will help the family to understand.” (Provider)
	Using well-child visits to introduce research opportunities	“I think the they were primarily well child visits and honestly that's also the majority of my schedule because I'm not in clinic all that often so most of my visits are physicals.” (Provider)
		“For an urgent visit, I'm probably asking a lot about these things, but I would probably be less likely to [recruit] if it was a patient, I was seeing for an asthma exacerbation who is not my patient. (Provider)
*Strategies for research teams to support pediatric practices in clinical trial recruitment*	Consider potential fit between research opportunity and pediatric practice sites	“Really important for it to meet your interests. I think that’ the most important thing … I don't know how much I would have been involved in this if I wasn't interested in the program” (Provider)
		“We have a scholarly outlook at our, mission here, you know, or their mission driven as part of as employees at the medical school and teachers in the medical school” (Provider)
		“I think everyone just so stretched that offering something that's meaningful to that individual is really important that. That meaningfulness can vary from individual to individual … for someone, it may be FTE, one, you know, one less day in clinic. For other, it's to get a co-author to be able to try to help them in their promotion process moving forward.” (Provider)
		“It depends upon whether for me, I mean, if it's something esoteric, I'm not interested. If it's something really gonna impact my patients and make a difference, why not? Again, If it's going to be a lot of work for my staff to do, my answer has always been no” (Provider)
	Support pediatric practices in identifying eligible patients	“I think the way that the program handled it was maybe easy for us to do, because you know, a lot of it, the back work was already being done for us and I didn't have to spend a lot of time, you know, looking to see who was affected and who would be enrolled into the program.” (Provider)
		“We hooked them up to join get on our system from their offices [to help us identify eligible patients]. It was just streamlined. It was so streamlined, it made it easy to do and I enjoyed it”” (Provider)
	Ensuring research team is accessible to pediatric practice staff throughout study	“If it hadn't been for [research staff involvement] I don't know how successful this would have been in my clinic” (Provider)
		“I remember like [the research coordinator] was like super accessible to questions and stuff like that. A few times I know I like emailed her and texted her back and forth. And that amount of access was crucial.” (Provider)
		“And [the research coordinator] made a lot of these calls as well. She was wonderful. She was great and helpful. But that helped a lot.” (Practice Manager)
	Funding and/or protected time for research duties	“Having funds to have research assistants. Having funding to carve out some of our clinical time so that we could devote time to doing research instead of having to do it on the free time that we have.” (Provider)
		“If there is a time commitment where we would need to have somebody do extra work, maybe some compensation for the time would be appreciated.” (Provider)
	Ensuring that research teams align with children and families in terms of racial/ethnic make-up and relatability	“We are dealing with a lot of our patients who are very low income and this woman would come in [for recruitment] with her coach bag … and it was very intimidating to our patients, you know, because they couldn't identify with them” (Practice Manager)
		“[Seeing] other people, if these families see others who look like them or practice the same cultural values as them recruiting.” (Provider)
		“[Those recruiting don't] look like those racialized, minoritized patients and families … and there are different levels of power dynamics” (Provider)
		“I think the vast majority of our trials have excluded people who don't speak English.” (Provider)
	Considering how to mitigate socioeconomic barriers to participation for historically marginalized families	“Incentives or transportation assistance would also help— “75–80 % Medicaid practice and the people are financially strapped and limited” (Provider)
		“Some people don't have computers. Some people don't have transportation.” (Provider)

### 3.1 considerations for recruitment of historically marginalized children and families into trials in pediatric practice settings

Pediatric practice staff including pediatricians and nurses noted using several approaches when offering clinical trial opportunities to historically marginalized children and families: (1) openly discussing with families the potential mistrust in research to help build trust in research processes, (2) discussing the potential clinical benefits and financial compensation from participating in research and (3) using well-child visits to introduce research opportunities. Many interviewees reported that in their experience and interactions with longstanding patients they heard that mistrust in research is pervasive. They inferred that such mistrust may be acting as a barrier to recruitment, particularly for historically marginalized children and families. When mistrust can be briefly addressed by the pediatric practice staff who have existing relationships with the family, this can build trust in research opportunities, particularly for families that may otherwise be reluctant to participate. Relatedly, practice staff can discuss the possible benefits of research participation on the child's health and note that if they participate, they will be compensated for their time. Finally, several practice staff noted that well-child visits afforded more time to discuss research opportunities and thus believed this to be the optimal time for introducing research opportunities to families.

### 3.2 Strategies for research teams to supportpediatric practices in clinical trial recruitment

Pediatric practice staff also reflected on how research teams can better facilitate successful recruitment efforts within pediatric practice settings which serve historically marginalized children and families. Many participants reflected on the need for clinical trial opportunities to be a good alignment within their practice in terms of provider and staff interest in the topic, belief that the trial will be beneficial to their patients and families and fit within their existing clinical workflow. Additionally, several ways through which research staff can support pediatric practice staff once recruitment has started emerged: (1) support identifying potentially eligible children and families by running reports using electronic medical record systems, (2) ensuring research team is accessible to pediatric practice staff throughout study, (3) providing funding and/or protected time for practice staff to conduct research duties, 4) ensuring that recruitment efforts align with children and families in terms of racial/ethnic make-up, language, and relatability, and (5) considering how to mitigate socioeconomic barriers to participation for historically marginalized families. Several practice staff emphasized that the specific individual who presents the research opportunity and speaks to families about recruitment is important to consider. For example, language and cultural concordance between the person presenting the research and the family would be ideal. However, as one staff member noted, at the very least the individual presenting the research has to understand the family's background and be able to form a connection with that family. Finally, for many of these families, practice staff noted that there are socioeconomic barriers that may hinder families' ability to participate in clinical trials so research teams should work to overcome these barriers. Several staff members highlighted that transportation may be a challenge for many of these families and that conducting recruitment and subsequent study assessments via the phone or video conferencing may therefore be preferable for these families.

## 4 Discussion

Clinical trial recruitment, particularly efforts focused on historically marginalized children and families, is inherently complex. Through interviews with pediatric practice staff who participated in the recruitment of children and families from low income and historically marginalized backgrounds into a community-based pediatric asthma trial [20], we identified several strategies that can be used by researchers and practice staff to support these efforts. Our findings highlight potential practical steps that can be taken by pediatric practice staff and research teams to support diverse trial recruitment, all of which our team is currently using to promote recruitment of minoritized children and families in our large, randomized control trial.

Through interviews, we learned that pediatric practice staff think it is important to consider the cultural and linguistic fit between individuals doing recruitment and the target population. Many participants described examples of recruitment going well when families had similar cultural backgrounds as the provider or staff member conducting recruitment. These findings align with those of others who call for the importance of cultural concordance between those conducting recruitment and potential participants to promote diversity in clinical trials [25,26]. To support this, research teams could focus on identifying clinics that have providers and staff who may be able to communicate with children and families in their preferred languages or have staff from similar cultural backgrounds. Moreover, as several providers in our study noted, and as has been highlighted in others [27], limited availability of multilingual research study materials can be a barrier to recruitment of historically marginalized children and families. To address this, researchers could consider devoting funds to allow for expansion of inclusion criteria beyond English-language speaking and provide study materials in multiple languages.

In this work, nearly all practice staff noted the importance of logistical support from the research team leading up to and throughout the study and recruitment period to support the identification of eligible patients. Participants suggested that research teams could help facilitate or generate reports using electronic medical records to identify potentially eligible children who could then be offered participation in the trial. We believe this may be particularly important for recruiting historically marginalized populations who often miss well-child visits [28], as participants in our study reported that the majority of their recruitment efforts are conducted during these visits. Moreover, pediatric practice staff also expressed an appreciation for the time the research team devoted to supporting practices during recruitment as well as an appreciation for any compensation or stipend provided to cover the practice's time. Providing this level of logistical support has been noted in other studies as aiding recruitment efforts in practices [29,30].

Finally, related to the actual recruitment conversation several notable findings emerged regarding strategies that pediatric providers could consider using: introducing research studies at well child visits, directly addressing history of mistrust in research in conversations with potential participants, and thoroughly explaining the potential health benefits of research participation for children who participate in trials (e.g., access to innovative interventions, close monitoring of chronic health conditions). For many of our participants, well child visits emerged as the best option to conduct recruitment conversations as they allowed more time for our discussion. To our knowledge this has yet to be described as a strategy to facilitate trial recruitment, but merits further exploration, particularly to assess receptivity of families to these conversations during well child visits. Moreover, many participants in our study noted that based on their years of experience and interaction with families in their clinics, they believe mistrust in research is a barrier to recruitment of historically marginalized children and families. It is well documented that lack of trust in providers, the medical system, and researchers and this is a barrier to clinical trial recruitment. [31]. Mistrust could be attributed to a multitude of factors including but not limited to historical inquiries, racism in medicine, individual staff actions, and health literacy [32,33]. Participants in our study reported that, from their perspective, this mistrust may be due to inherent power dynamics between families and providers, history of medical racism, and low health literacy. There is an urgent need for work to be done by researchers and providers alike to combat lack of trust in research and address the socioeconomic factors faced by historically marginalized children and families as barriers to research participation. Finally, participants in our study noted that discussing both clinical benefits *and* financial incentives of trial participation is critical. For some families these financial incentives may mitigate socioeconomic barriers and allow them to participate in research activities that they otherwise could not have. Several providers commented that incentives or transportation assistance would be helpful, particularly given that most of their patient population is on Medicaid and faces multiple socioeconomic barriers. This echoes previous work finding that these are important topics to cover during recruitment [8,34]. However, the impact of offering financial incentives to facilitate recruitment of historically marginalized populations is mixed [35,36] and is likely complicated by some of the power dynamics and barriers noted above. Though, none of our participants spoke to this and nearly all reported that financial incentives could be helpful.

### 4.1 Strengths and limitations

The primary strengths of this study are the inclusion of pediatric practice staff from both community and academic practices. Moreover, this study is the first to our knowledge that qualitatively explores pediatric practice staff perspectives on strategies that promote successful recruitment of diverse children into research studies. Several limitations should be noted. Pediatric providers and clinic staff were interviewed over a year after initial trial recruitment efforts and thus recall bias may have impacted their responses. Additionally, there is the possibility that those who did not participate could have had different perspectives than those captured from staff members who did participate. Additionally, we recruited staff participants from practices in a single, mid-sized city and their experiences may not reflect those of pediatric staff working in other geographic locations. Finally, we would like to acknowledge that the goal of these interviews was to ascertain pragmatic, real-world solutions to address recruitment challenges for historically marginalized children and families and thus, due to the brief nature of the interviews, we did not participants’ perspectives on how historical context of racism in medicine and research may be contributing to inequities, though this certainly warrants further in-depth exploration.

## 5 Conclusion

While the importance of diversity in clinical trials is becoming more recognized, children and families from historically marginalized backgrounds remain underrepresented in research hindering the advancement of child health equity. While recognizing the many complex dynamics that affect trial recruitment for these populations, we sought to identify concrete strategies that could be used by both research teams and pediatric practices to support improved diversity in pediatric clinical trials. Research teams can make efforts to ensure recruitment sites are well-prepared and supported to conduct recruitment, especially of historically marginalized populations. Pediatric practice staff can utilize well child visits to present research opportunities, discuss mistrust in research with families and explain the potential clinical benefits of and financial compensation from participating in research. While these approaches alone will not be sufficient to fully alleviate inequities, we believe their use can help support greater inclusion of children and families from historically marginalized backgrounds within clinical trials.

## CRediT authorship contribution statement

**Janvi D. Nanavati:** Writing – review & editing, Writing – original draft, Methodology, Formal analysis. **Daniel Mendoza Martinez:** Writing – review & editing, Writing – original draft, Methodology, Formal analysis. **Grace W. Ryan:** Writing – review & editing, Writing – original draft, Supervision, Methodology, Formal analysis, Conceptualization. **Melissa Goulding:** Writing – review & editing. **Sonia Radu:** Writing – review & editing. **Michelle Spano:** Writing – review & editing, Supervision, Project administration. **Ted Kremer:** Writing – review & editing. **Kali Pereira:** Writing – review & editing. **John Almeida:** Writing – review & editing. **Christine Frisard:** Writing – review & editing. **Sybil Crawford:** Writing – review & editing. **Milagros C. Rosal:** Writing – review & editing. **Nancy Byatt:** Writing – review & editing. **Stephenie C. Lemon:** Writing – review & editing. **Lori Pbert:** Writing – review & editing. **Michelle Trivedi:** Writing – review & editing, Supervision, Methodology, Formal analysis, Conceptualization.

## Funding sources

This work was supported by the 10.13039/100000050National Heart, Lung, and Blood Institute. The funder was not involved in study design, data collection, analysis or interpretation, writing of this manuscript, or decision to submit this manuscript for publication.

## Declaration of competing interest

The authors declare the following financial interests/personal relationships which may be considered as potential competing interests: Dr. Byatt has received salary and/or funding support from Massachusetts Department of Mental Health via the Massachusetts Child Psychiatry Access Program for Moms (MCPAP for Moms). She is also the Medical Director of Research and Evaluation for MCPAP for Moms and the Executive Director of the Lifeline for Families Center at UMass Chan Medical School. She has served as a consultant for The Kinetix Group, VentureWell, and JBS International.

## Data Availability

Data will be made available on request.
