# Supplementary material for: Promoting the recruitment of historically underrepresented children and families in clinical trials: Perspectives of pediatric clinic staff
Source: Contemp Clin Trials Commun. 2026 Jan 27;49:101607. doi: 10.1016/j.conctc.2026.101607 (PMC12874319; doi:10.1016/j.conctc.2026.101607)
Supplement: Multimedia component 1 [file mmc1.docx]

**Supplementary Materials: Interview Guide:**

**Questions:**

1. Can you tell me a little bit about how you had the conversation with families about the study?
   1. If not you/beyond you, who at your practice was doing this/involved in this?
   2. Probes:
      1. When were you having these conversations with families? At well child visits? Other types of appointments?
2. What you think motivates children/families in your practice to participate in research studies?
   1. Probes:
      1. Can you think of any specific examples from families you talked to about the Asthma Link study?
      2. Did families express any concerns about being in the study? Could you tell me a little bit about that?
      3. Do you think incentives would help?
3. Aside from this, are you/your practice currently participating in any other research programs like this?
   1. Briefly, can you tell me a little bit about what your role is in them?
4. What motivates you to participate in research efforts?
   1. Probes:
      1. What kids of research/programs like this do you prioritize participating in?
      2. How do you/your office decide what’s “worth” participating in?
5. Generally, what would be barriers to you/your clinic participating in research or clinical trial activities like this?
6. Similarly, what kinds of things would make it easier for you/your practice to participate in research activities?
7. It is also important to us that we have representation from diverse children and families in research studies so we can examine interventions that help them. Fortunately in this Asthma research we have done so….are there things you think that help make this possible (to help encourage underrepresented minority children/families to participate in research)?
   1. Probes:
      1. Some examples might be additional support with translation? Are there specific types of incentives you think might be helpful?
      2. Specific ways to talk to these families about research?
8. Finally, is there anything else that you want to share that is relevant to this conversation on recruitment for/participation in research activities?
